# Novel imaging technologies for genetic diagnoses in the inborn errors of metabolism

**DOI:** 10.20517/jtgg.2020.09

**Published:** 2020-11-13

**Authors:** Andrea L. Gropman, Afrouz Anderson

**Affiliations:** 1 Department of Neurology, Children’s National Medical Center, Washington, DC 20010, USA.; 2 Department of Research, Focus Foundation, Crofton, MD 21035, USA.

**Keywords:** Genetics, inborn error of metabolism, MRI, Magnetic Resonance Spectroscopy, functional near infrared spectroscopy, functional MRI, diffusion tensor imaging, neuroimaging

## Abstract

Many inborn errors of metabolism and genetic disorders affect the brain. The brain biochemistry may differ from that in the periphery and is not accessible by simple blood and urine sampling. Therefore, neuroimaging has proven to be a valuable tool to not only evaluate the brain structure, but also biochemistry, blood flow and function. Neuroimaging in patients with inborn errors of metabolism can include additional sequences in addition to T1 and T2-weighted imaging because in early stages, there may be no significant findings on the routine sequnces due to the lack of sensitivity or the evolution of abnormalities lags behind the ability of the imaging to detect it. In addition, findings on T1 and T2-weighted imaging of several inborn errors of metabolism may be non-specific and be seen in other non-genetic conditions. Therefore, additional neuroimaging modalities that have been employed including diffusion tensor imaging (DTI), magnetic resonance spectroscopy, functional MRI (fMRI), functional near infrared spectroscopy (fNIRS), or positron emission tomography (PET) imaging may further inform underlying changes in myelination, biochemistry, and functional connectivity. The use of Magnetic Resonance Spectroscopy in certain disorders may add a level of specificity depending upon the metabolite levels that are abnormal, as well as provide information about the process of brain injury (i.e., white matter, gray matter, energy deficiency, toxic buildup or depletion of key metabolites). It is even more challenging to understand how genetic or metabolic disorders contribute to short and/or long term changes in cognition which represent the downstream effects of IEMs. In order to image “cognition” or the downstream effects of a metabolic disorder on domains of brain function, more advanced techniques are required to analyze underlying fiber tracts or alternatively, methods such as fMRI enable generation of brain activation maps after both task based and resting state conditions. DTI can be used to look at changes in white matter tracks. Each imaging modality can explore an important aspect of the anatomy, physiology or biochemisty of the central nervous system. Their properties, pros and cons are discussed in this article. These imaging modalities will be discussed in the context of several inborn errors of metabolism including Galactosemia, Phenylketonruia, Maple syrup urine disease, Methylmalonic acidemia, Niemann-Pick Disease, type C1, Krabbe Disease, Ornithine transcarbamylase deficiency, Sjogren Larsson syndrome, Pelizeaus-Merzbacher disease, Pyruvate dehydrogenase deficiency, Nonketotic Hyperglycinemia and Fabry disease. Space constraints do not allow mention of all the disorders in which one of these modalities has been investigated, or where it would add value to diagnosis or disease progression.

## INTRODUCTION

Many inborn errors of metabolism (IEMs) are associated with an acute onset of progressive symptoms and irreversible brain injury^[1–5]^. Research from preclinical models is delving into the etiologies of how a metabolite intoxication accounts for the specific cognitive and neurologic findings observed in IEM patients related to the time of injury or many years later^[6,7]^. IEM-associated brain injury patterns may be region specific and primarily cause gray matter or white matter damage, but typically is more complicated and may include damage to neurons and the supporting glia at the same time or later in the course. In children, this occurs on the backdrop of development of some of the very systems that are impacted later, such as executive function^[8]^.

It is not clear why a global insult such as IEM may cause more selective damage to particular sets of cells. This could involve deep gray matter neurons, white matter, the putamen (and to a lesser degree, the globus pallidi) as seen in glutaric aciduria type 1^[9]^, putamen in certain mitochondrial cytopathies^[10]^, or the globus pallidus in methylmalonic acidemia^[11]^. There may be damage to regions that share a particular neurotransmitter system. This remains poorly understood and often requires preclinical investigations.

Neuroimaging has already shown great potential for the investigation and management of IEM and other genetic conditions. Given its non invasive nature, it allows for longitudinal assessments and follow-up. Each modality may contribute something unique about the timing or pathology, and can be used as a biomarker to study the disease course or therapeutic intervention. One criticism has been that the findings on routine T1 and T2-weighted imaging are nonspecific and may not allow differentiation between disorders. In those cases, additional magnetic resonance images (MRI) sequences and/or modalities enables a higher diagnostic specificity. Beyond establishing a diagnosis, MRI may be used to understand pathology, disease progression and long term impact of the disease on higher cognitive function with the use of functional MRI (fMRI) and functional near infrared spectroscopy (fNIRS). The latter techniques are important as therapy may lead to a decrease in a blood metabolite, yet there may be an elevation of a toxic compound in the CNS and the impact of damage on cognition and function may not be fully understood. These advanced modalities are not available in every hospital. However, the purpose of this review is not to mandate use of certain imaging modalities, but rather to highlight when certain imaging sequences may be useful to further understand the pathogenesis of a metabolic disorder. In addition, due to space constraints, it will not be possible to list every condition and its imaging characteristics. Rather, specific disorders will be highlighted in which recent literature has suggested the value of MRI in these conditions. Lastly, research into practice will use examples as to how multimodal imaging is applied in the clinical setting, leading to improved clinical management.

The term “neuroimaging” describes sequences beyond T1 and T2 weighted imaging. Most clinical routine images at major medical centers include a fluid attenuation inversion recovery (FLAIR). Some centers are able to reformat a high resolution T1 weighted image and perform volume analysis using voxel-based morphometry (VBM) to measure differences in gray and white matter volumes. To understand the cognitive outcomes of IEMs, one can use fMRI to study the neural networks that underlie cognitive pathways^[12]^. Another modality that can also give information about neural networks is fNRIs which is a less invasive and portable imaging modality (described below).

## MAGNETIC RESONANCE IMAGING, HOW IT WORKS

MRI works by exploiting differences in water proton spins between tissue types (gray matter, white matter, and cerebral spinal fluid) after the tissue is exposed to a radiofrequency pulse in a strong magnetic field. The subject’s head is enclosed in a head coil, and there is a body coil at the bed of the scanner. The coil serves as an antenna, converting electromagnetic waves into an electrical current, which is used to reconstruct the three dimensional images.

Despite its utility as both a research and clinical tool, routine MRI detection is limited to macroscopic alterations in brain structure. It lacks the spatial resolution to provide information regarding microstructural neuropathology, and does not capture dynamic processes in space and time related to brain function and metabolism. Additionally, many macrostructural neuropathological phenotypes lag behind the presentation of associated clinical manifestations until there is critical amount of macroscopic damage that can be seen on an image.

The advantages of MRI over CT include the lack of ionizing radiation, the ability to image in three orthogonal planes and the ability to better visualize the brainstem and cerebellum which are difficult to see in CT due to beam hardening artefacts. MRI is superior to CT in the evaluation of white matter pathologies. This is at the expense of longer imaging sequence times, need for sedation in children and sometimes lack of accessibility in an emergency situation.

## DIFFUSION TENSOR IMAGING

### How DTI works

DTI is an imaging technique in which contrast is based on differences in the diffusion of water molecules^[13]^. As a result, maps of white matter fibers can be generated. Since water diffusion in cells corresponds to cell geometry in axons, diffusion MRI can also be used to make inferences about white matter architecture. One standard measure is anisotropy which refers to the property of being directionally dependent. Three measures of diffusion in tissue can be quantitated using DTI: magnitude (ADC), orientation of diffusion, and degree of anisotropy (orientation of diffusion and deviation from uniform diffusion in all directions). The more unrestricted the water molecules are in their movement, the higher the ADC and the lower the anisotropy. DTI has in some cases shown to have predictive ability for example, in recovery from a traumatic brain injury^[14]^.

Understanding white matter integrity is important because underlying changes in neural networks are often aberrant white matter tracks. There are many well defined neural networks that play a role in cognitive functions such as working memory, attention and cognitive flexibility^[15]^. There is a rich literature that has focused on alterations in executive function, for example on large cohorts of children and adults with attention deficit disorders, Alzheimer disease, autism and post traumatic brain injury to name a few. Only recently has this been a topic of interest in inborn errors of metabolism.

## GALACTOSEMIA

Doyle *et al*.^[16]^ used a cognitive function battery demonstrating low scores for verbal and performance IQ, as well as low scores for memory and executive function. Subsequently, using diffusion tensor imaging, others found that compared to age- and gender-matched controls, patients with galactosemia had reduced volume in the left cerebellar white matter, the bilateral putamen, and left superior temporal sulcus. There was also lower fractional anisotropy and higher radial diffusivity values in the dorsal and ventral language networks compared to the controls^[17]^.

## PHENYLKETONURIA

Phenylketonuria (PKU) is perhaps the best studied metabolic condition in terms of MRI imaging. The underlying neurobiological aspects of PKU include deficits in executive dysfunction. While not completely understood, it is believe to be related to the often seen myelination abnormalities. DTI studies have identified restricted diffusivity in individuals with PKU across a number of brain regions, including the centrum semiovale, posterior-parietal occipital cortex, prefrontal cortex, optic radiation, putamen, and anterior corpus callosum^[18–25]^. Gross WM abnormalities seen at autopsy and on T1 and T2-weighted magnetic resonance images (MRI) have been reported in patients with PKU^[26–36]^ and white matter (WM) changes are evident by the second decade of life.

Subtle executive function deficits especially in inhibitory control have been reported in patients with phenylketonuria (PKU), and this is seen despite early dietary restrictions^[19,37]^. WM alteration was indeed influenced by life-long metabolic control but the severity score of WM alterations did not correlate with IQ or Executive function scoring^[35,36]^.

Maple syrup urine disease (MSUD) is a disorder involving the catabolism of branched-chain amino acids (BCAA; leucine, isoleucine, and valine) and is caused by deficiency of the branched-chain ketoacid dehydrogenase enzyme^[37,38]^. Two types of brain edema have been described in MSUD. Intramyelinic edema affects the myelinated white matter (cerebellar white matter, dorsal brainstem, cerebral peduncles, posterior limb of the internal capsule, and peri-rolandic cerebral white matter), thalami, and globus pallidi. Intramyelinic edema or “MSUD edema” is the more recognizable pattern, described as hyperintense on T2-weighted images, diffusion weighted imaging (DWI) and hypointense on T1-weighted images. DTI shows alterations in both ADC and fractional anisotropy (FA) values^[39–42]^. Intramyelinic edema which is believed to be a consequence of energy failure leads to the accumulation of water molecules that intercalate between the myelinic lamellae resulting in the splitting of the myelin layers. This can be detected by DTI (decreased FA values)^[41,42]^. The end result of this process may be delayed myelination and white matter atrophy with the aforementioned cognitive sequelae.

## METHYLMALONIC ACIDEMIA

DWI has been used to investigate ADC in methylmalonic acidemia (MMA). It has been reported to be associated with restricted water diffusion in the globus pallidus that may resolve after clinical interventions including carnitine supplementation. Findings from 12 MMA patients demonstrated significant reductions in FA in the globus pallidus, frontal, temporal and occipital white matter using DTI and we’re not appreciated on conventional T1/T2 images^[43]^. This suggests that, in addition to restricted water diffusion in the globus pallidus, MMA is associated with more widespread disturbances in white matter integrity. This is an example of how DTI proved superior to routine imaging in identifying these diffuse lesions. Neurocognitive lesions in MMA have yet to be investigated with fMRI.

## NIEMANN-PICK DISEASE, TYPE C1

Niemann-Pick disease, type C1 (NPC1), is a rapidly progressive neurodegenerative disorder characterized by cholesterol sequestration within late endosomes and lysosomes. There are no specific or reliable imaging markers that can be used for management and prognostication. Cerebellar volume deficits are found to correlate with disease severity. Diffusion tensor imaging (DTI) of the corpus callosum and brainstem, has demonstrated abnormalities with microstructural disorganization that has shown some association with the degree of NPC1 severity^[44]^.

## KRABBE DISEASE

Studies using DTI have shown that radial diffusivity and fractional anisotropy are sensitive in vivo as biomarkers of white matter microstructural damage in this condition. Using DTI, early white matter injury has been detected in asymptomatic neonates with Krabbe. The DTI metrics have shown to correlate with motor and cognitive functions after hematopoietic stem cell transplantation (HSCT), and as a marker of white matter development^[45]^.

## ORNITHINE TRANSCARBAMYLASE DEFICIENCY

MR imaging in patients with ornithine transcarbamylase deficiency (OTCD) is often normal in late-onset disease, heterozygotes, or in those not in hyperammonemic crisis. Gropman *et al.*^[46]^ used DTI to study adults with OTCD. In all cases, DTI proved more sensitive than T2-weighted imaging for detecting abnormalities in normal-appearing white matter. The extent of abnormality in white matter in turn correlated with cognitive deficits. The location of the deficits in the frontal white matter correlated with pathways that subserve executive function, attention, and working memory which are impaired in this patient population^[46]^.

Given that the goal of therapy in IEMs is to decrease morbidity to the brain and other organs, understanding the impact of these disorders on brain injury and remodeling can help frame treatment and monitoring in patients. Differential neural networks underlying a cognitive process may arise due to injury to the native pathway, and/or development of an accessory pathway over time. Since T1 and T2 weighted changes in white matter tracts are only sensitive to significant, macroscopic damage, quantitative, early microscopic features of myelination and axonal integrity can be gleaned by using DWI and DTI which are used to study microstructural variance in white matter fiber tracts^[46]^.

## MAGNETIC RESONANCE SPECTROSCOPY

Magnetic resonance spectroscopy (MRS) is an imaging technique used to measure brain metabolism^[47]^. It is popular in IEMs for monitoring of disease progression and therapeutic response.

MRS is noninvasive. Its use allows one to gain information relevant to tissue biochemistry and metabolism^[48]^. MRS is performed with the same hardware used for anatomical imaging. Instead of an image, a spectrum is produced. The area under the peaks is proportionall to the concentration of the relevant metabolite. MRS studies of humans and genetically altered animals are feasible as are studies of cultured cells, histological tissue samples, and chemical extracts of tissues^[49]^. A benefit of MRS is the lack of ionizing radiation. Therefore repeated scanning of patients including infants and children is feasible and acceptable. However, MRS is very sensitive to movement and the peaks can become wide and uninterpretable which makes it a difficult imaging modality to perform in unsedated children.

MRS allows measurement of chemicals in the brain. It is based on the principles of nuclear magnetic resonance. Magnetic resonance reveals the interaction between a molecule and an external magnetic field.

The nuclei most relevant to complex human conditions are ^1^H, ^31^P, ^13^C, ^7^Li, and ^19^F. Those most studied include hydrogen (^1^H) (also referred to as proton MRS) and phosphorus (^31^P). (^13^C) imaging is cumbersome but can be useful to study flux through the citric acid cycle which is relevant for many of the inborn errors of metabolism.

In order for MRS to effectively measure the key metabolites, the molecules must be relatively mobile within the tissue. Large macromolecules (i.e., molecular weights greater than 20,000 Daltons) do not show enough free movement and cannot be detectedd. There must also be a large enough concentration of atomic nuclei of a specific type to generate a recognizable signal/noise ratio in the millimolar range concentration.

**Figure 1.**
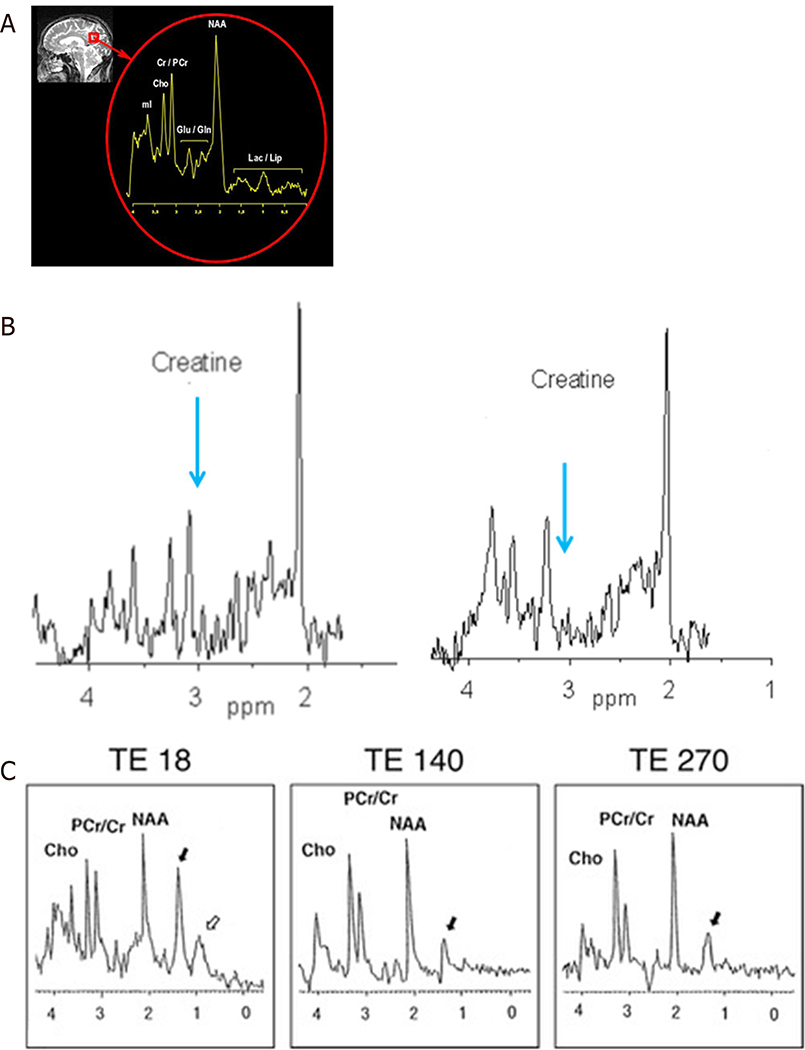
Demonstration of normal ^1^H MRS (A); there is an absent creatine peak (arrow, second panel). The first panel shows the normal creatine peak (B); and Sjogren Larsson syndrome demonstrating a prominent lipid peak (C). Adapted from Mano *et al*.^[50]^. Lac/lipid: lactate and lipid peaks; NAA: N-acetylaspartate; Glu/Gln: glutamate and glutamine; Cr/PCr: creatine and phosphocreatine; cho: choline; mI: myoinositol

The peak areas correspond to the number of nuclei and are extrapolated to concentration. Proton MRS is the most widely used spectroscopy technique for the brain^[49]^. The most common chemicals studied are N-acetyl-L-aspartate (NAA), total creatine (tCr) which is a sum of creatine (Cr) and phosphocreatine (PCr), choline (Cho), myo-inositol (mI), lactate (Lac), glutamate (Glu), and glutamine (Gln), known on low field MRI as “Glx”. In addition, amino acids, lipid, and gamma-aminobutyric acid (GABA) may be detected; but require editing. The number of metabolites seen will depend upon the chosen echo time (TE) and field strength with more metabolites seen on short TE. Long TE MRS is sufficient to evaluate lactate (if present), NAA, creatine, and choline Glu. Gln and mI are not detectable at long TE due to short relaxation times. These common chemicals can provide information about disease states: NAA: mitochondrial oxidative metabolism and marker for neuronal viability. It is also the source of acetyl groups for lipid synthesis; Cr and phosphocreatine (PCr): markers of creatine to phosphocreatine energy conversion; Cho: precursor for the neurotransmitter acetylcholine and membrane phospholipids, phosphatidylcholine, and sphingomyelin; mI: neuronal signaling molecule of the phosphoinositide pathway and osmoregulation; Lac: marker of anaerobic metabolism and Glu and Gln: neurotransmitters in the CNS. A number of MRS studies have been conducted in IEMs and are shown in the table [Table 1]. Figure 1A shows a normal MRS, and Figure 1B and C show spectra with missing or extra peaks. A few disorders are highlighted below.

## GLUTARIC ACIDURIA TYPE I

Acute striatal necrosis is a devastating consequence of the encephalopathic crisis seen in patients with glutaric aciduria type I (GA-I). Insidious-onset patients have been shown to have a latency phase of 3.5 months to 6.5 years between detection and clinical manifestation of dorsolateral putaminal lesions^[51]^. MR spectroscopy showed a decreased N-acetyl-aspartate (NAA) peak and NAA/creatinine (Cr) ratio at the basal ganglia in three encephalopathic patients. The values of NAA/Cr ratios in these patients were below that of controls (range: 0.97–1.12 *vs*. 1.61–1.97). In patients with motor symptoms, MRI showed abnormalities in FLAIR and T2-weighted sequences. There was bilateral and symmetrical hyperintensity in the putamen, caudate, globus pallidus and cerebral peduncles.

## MAPLE SYRUP URINE DISEASE

MRS has also been used in maple syrup urine disease (MSUD). The most specific biomarker is elevation of leucine, isoleucine and valine and corresponding 2-oxo acids. This corresponds to a peak at 0.9 ppm on MRS. Heindel *et al*^[52]^ reported a nine-year-old patient who suffered acute metabolic decompensation. The elevated peak at 0.9 ppm was seen in the early phase of disease, during decompensation and normalized after recovery^[53]^.

## PELIZEAUS-MERZBACHER DISEASE

Pelizeaus-Merzbacher disease (PMD) is a hypomyelination disorder due to a primary defect in myelin formation^[54]^. Delayed and low myelin is appreciated on MRI^[55]^. Pizzini *et al.*^[56]^ have observed reduction in NAA and mild increase in choline levels in the affected white matter. Sener noted a reduction in Cho/Cr^[57]^.

## METHYLMALONIC ACIDEMIA

Methylmalonic academia (MMA)^[58]^, an autosomal recessive disorder of amino acid metabolism occurs due to inability to convert methylmalonic acid to succinic acid. MMA results from mutation of the methylmalonyl-CoA mutase apoenzyme, or its coenzyme adenosyl cobalamin^[58]^. MMA typically presents in early infancy. Neonatal and infancy onset presents with vomiting, lethargy dehydration and metabolic acidosis. Seizures, hypotonia, intellectual disability, chorea, developmental delay, oral motor dyspraxia and metabolic decompensation secondary to infection are key clinical features. These patients are at risk of metabolic stroke which appear as T2-weighted signal abnormalities in the globus pallidus of the basal ganglia on MRI^[59,60]^. These strokes may unmask a movement disorder manifest by chorea, dystonia, or athetosis. One case report has also provided evidence of reduced NAA and increased lactate signals on MRS which normalized with comprehensive clinical management^[61]^.

## PYRUVATE DEHYDROGENASE DEFICIENCY

Pyruvate dehydrogenase complex converts pyruvate to acetylcoenzyme A, which is necessary in TCA cycle energy production. Excessive pyruvate is converted to lactate. Pyruvate dehydrogenase defects can manifest with Leigh syndrome features on MRI^[62]^. On neuroimaging, deep gray nuclear T2-weighted signal abnormalities and white matter hyperintensity are typical. The corpus callosum can be dysgenetic^[63]^ since neurogenesis, migration, and neuronal organization need adequate energy production.

^1^H MRS can demonstrate lactate elevation; the absence of this finding does not exclude the diagnosis^[64]^. Creatine can be increased, but NAA and Cho are often normal. Myoinositol may be increased. A novel MRS peak at 2.37 ppm has been described, attributable to pyruvate. In some cases, an Acetate peak is seen at 1.9 ppm.

## NONKETOTIC HYPERGLYCINEMIA

Nonketotic hyperglycinemia (NKH) is caused by a glycine cleavage defect and manifests with seizures and hypotonia. Glycine is an excitatory neurotransmitter in the brain and inhibitory in the spinal cord. Glycine neurotoxicity may cause structural brain abnormalities in NKH^[65]^. MRI findings include symmetrical, hyperintense signal and reduced diffusion along the myelinated tracts, notably in the internal capsules, ventrolateral thalami, posterior putamen, dorsal brainstem, cerebellar peduncles, and deep cerebellum^[65]^. The corpus callosum is always abnormal and may be hypoplastic or dysgenetic^[66]^. Elevated glycine is found at 3.6 ppm and MRS helps with diagnosis. Intermediate or long TE MRS is necessary to delineate glycine from the myoinositol peak which overlap on a short TE spectrum.

## ORNITHINE TRANCARBAMYLASE DEFICIENCY

Ornithine Transcarbamylase deficiency (OTCD) is the most common among the six enzyme deficiencies in urea cycle disorders (UCD). OTC is a proximal enzyme of the urea cycle which is involved in the conversion of ammonia to urea. Ammonia is a highly neurotoxic moiety. Hyperammonemia (HA) can cause behavior changes, seizures and coma. One of the earliest changes on MRI is seen on MRS. A glutamine peak is observed due to elevation during acute HA, with a corresponding myoinositol reduction, presumably reflecting osmotic buffering^[67–69]^. OTCD disease severity correlates with glutamine and inversely correlates with myoinositol concentrations^[11]^. Reduction in choline occurs in patients with recurrent decompensation and longstanding HA.

## PET IMAGING

Positron emission tomography (PET) uses radioactive substances (tracers) to visualize and measure various metabolic processes in the body. PET is mainly used to measure metabolism, blood flow, regional chemical composition, and absorption^[70]^.

## FABRY DISEASE

Fabry disease (FD) is an X-linked lysosomal storage disorder, characterized by decreased or absent activity of the lysosomal enzyme alpha galactosidase A due to mutation of the alpha galactosidase A gene at Xq22.1. Clinical symptoms as a result of this accumulation include renal and cardiac failure, painful acroparaesthesias, angiokeratomas, hypohydrosis, corneal dystrophy, and stroke^[71–77]^. Strokes are seen in 25% of males and 21% of manifesting female carriers. PET imaging has been applied to Fabry disease to determine if it adds any sensitivity. In one study, MRI was deemed sufficient for monitoring and PET scanning with fluorodeoxyglucose (FDG-PET) does not add any further specificity^[78]^.

FDG-PET was used to measure regional glucose metabolic rate in patients with phenylketonuria (PKU) patients before and 4 months after sapropterin therapy. The study was limited by a small sample size (5 subjects) and none had responded to sapropterin therapy; defined as 30% decrease in blood Phe level. In addition, gglucose metabolism also appeared depressed in the cerebellum and left parietal cortex while elevated in the frontal and anterior cingulate cortices as well as in left Broca’s area and right superior lateral temporal cortex bilaterally^[79]^.

## FUNCTIONAL MRI

Roy and Sherrington first described neurovascular coupling in i890^[80]^ which forms the basis of MRI findings. Sherrington’s work showed that neuronal metabolism and cerebral blood flow are linked and this property is exploited to generate a blood oxygenation level-dependent (BOLD) signal in activated regions of the brain. These are then used to generate an activation map for each task the participant performs in the scanner. Active neurons consume glucose and oxygen, and this is restored by the local dilation of cerebral vessels with increase in oxygenated hemoglobin. This occurs during an energy demanding process: neurotransmission. The MRI pulse sequences are sensitive to the magnetic contrast between oxygenated and deoxygenated hemoglobin and that gives rise to the signal on the MRI. As a result, it can be used to map the hemodynamic response of local brain regions in relation to a cognitive, motor or resting state activity^[81]^.

After the study is completed, an activation map for a given task condition (working memory task, motor task, resting state, *etc*.) can be constructed. Analysis in fMRI involves averaging scores across multiple subjects to generate a group-wise image rather than individual subject maps. Statistical analyses are performed to assess BOLD signal differences between the experimental condition (i.e., task).

One of the limitations of fMRI, is the large number of subjects required to account for inter-subject variability in order to achieve an acceptable signal-to-noise ratio. Therefore, adaptations need to be made when studying rare disorders since the sample sizes are typically small. There has yet to be a consensus on using fMRI to probe neurocognitive phenotypes at the single subject level but it is useful in monitoring brain function at the group level.

## RESTING STATE FMRI

A recent development in neuroimaging research is the study of brain function in “resting” conditions, i.e., when the subject is at rest and not performing a task. There are several networks that operate at rest. The most widely studied resting state network (RSN) is the default mode network (DMN). DMN includes the precuneus/posterior cingulate cortex (PCC), mesiofrontal/anterior cingulate cortex (ACC), and temporoparietal areas^[82]^.

The term “default mode” was first used by Raichle in 2007^[83]^ to describe resting brain function. It has been associated with introspective thought^[84,85]^. Disruption of default mode has been linked to several disorders including Alzheimer disease and autism^[86]^. It is being explored in some IEMs.

PKU: fMRI in PKU has been robustly developed. Christ *et al*.^[87]^ have achieved the most in this regard and found the prefrontal cortex of PKU subjects had atypical neural activity during working memory performance even in those who were treated in childhood.

Resting state studies in PKU have demonstrated alterations in default mode, salience, and visual network. Patients showed alterations in networks involving the medial prefrontal cortex, parietal lobule and (pre) cuneus, which have been shown to underlie spatial orientation and attention^[88]^.

rsFMRI in galactosemia has shown alterations in activation of several important pathways including default mode, salience and visual networks. Patients showed alterations in networks underlying spatial orientation and attention, working memory, sensory-motor integration and motor speech planning^[89]^.

## TASK BASED FMRI

### PKU

Patients with PKU show deficits in inhibitory control despite early dietary treatment. Sundermann *et al*.^[34]^ used a Go-No Go task in young adult patients with PKU *vs*. control subjects and demonstrated more errors of commission and slightly increased activation associated with inhibitory control. This did not reach statistical significance.

### UCD

Patients with OTCD also experience impairment in frontal lobe function, especially in executive functioning, working memory, and motor planning. fMRI studies have been performed to evaluate functional connectivity of two resting-state networks, the default mode and set-maintenance--between OTCD patients and healthy controls, demonstrating reduced internodal functional connectivity in the DMN and set-maintenance network as a likely consequence of hyperammonemia^[90,91]^.

## FUNCTIONAL NEAR INFRARED SPECTROSCOPY

Most recently, several research groups have used fNIRS as a surrogate to fMRI imaging as it is portable, not subjected to the confines of the scanner and is not degraded by movement. Since subjects are awake in the scanner during fMRI experiments, fNIRS might prove to be an alternative method for subjects who are claustrophobic, cognitive impaired, or too young to cooperate in the MRI environment. fNIRS therefore have advantages for research on infants or more cognitively impaired individuals^[92–95]^.

**Figure 2.**
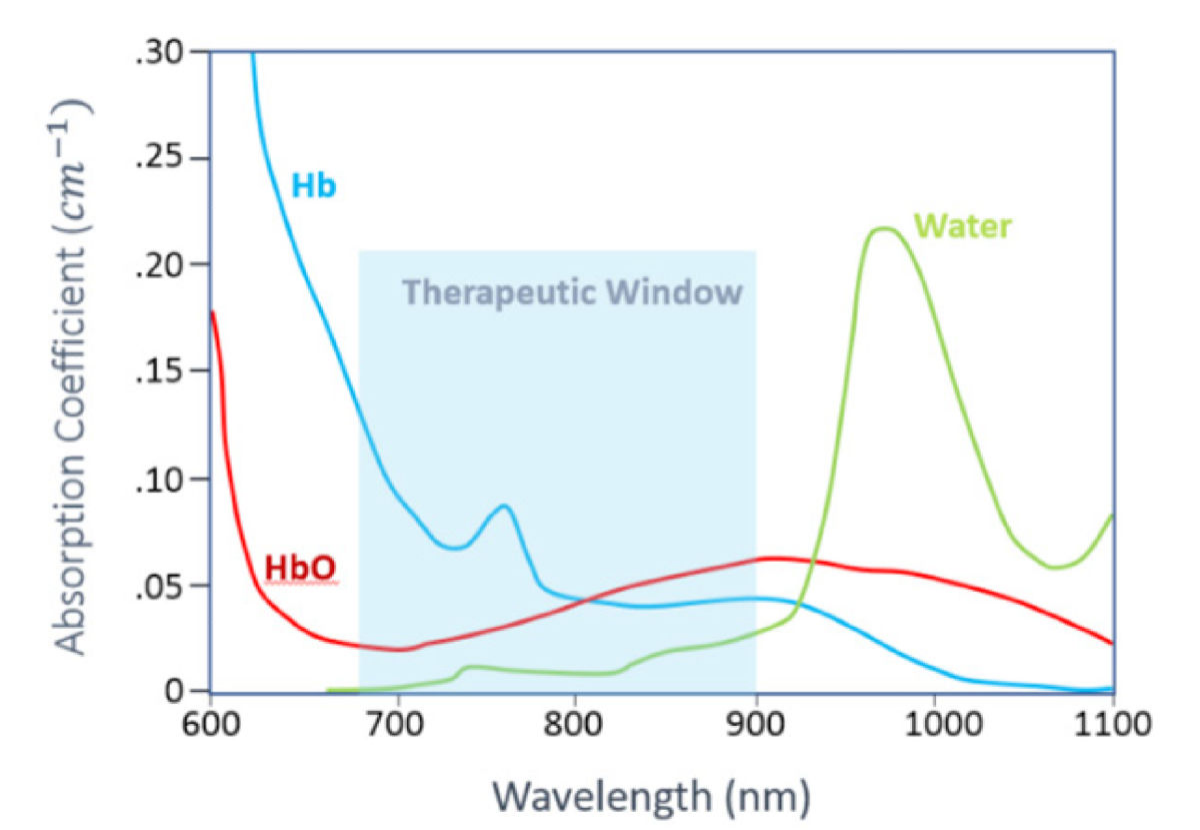
Absorption coefficient of Oxy-hemoglobin (HbO), Deoxy-hemoglobin (HbR) and water in the near infrared region

NIRS and fNIRS is an emerging technology for noninvasive measurements of cerebral hemodynamics associated with brain activity. It is not done in a scanner, and uses light in the range of 700 nm to 1000 nm. Compared to other well-established brain imaging modalities, such as fMRI and PET, this technique offers unique features with higher temporal resolution (in order of milliseconds). The status of oxyhemoglobin and deoxyhemoglobin changes can be measured. The instruments are small and are tolerant of subject motion which is often a limitation in imaging young and cognitively impaired subjects. fNIRS is based on the concept of diffuse optics to measure the hemodynamic response in cortical regions [Figure 2].

**Figure 3.**
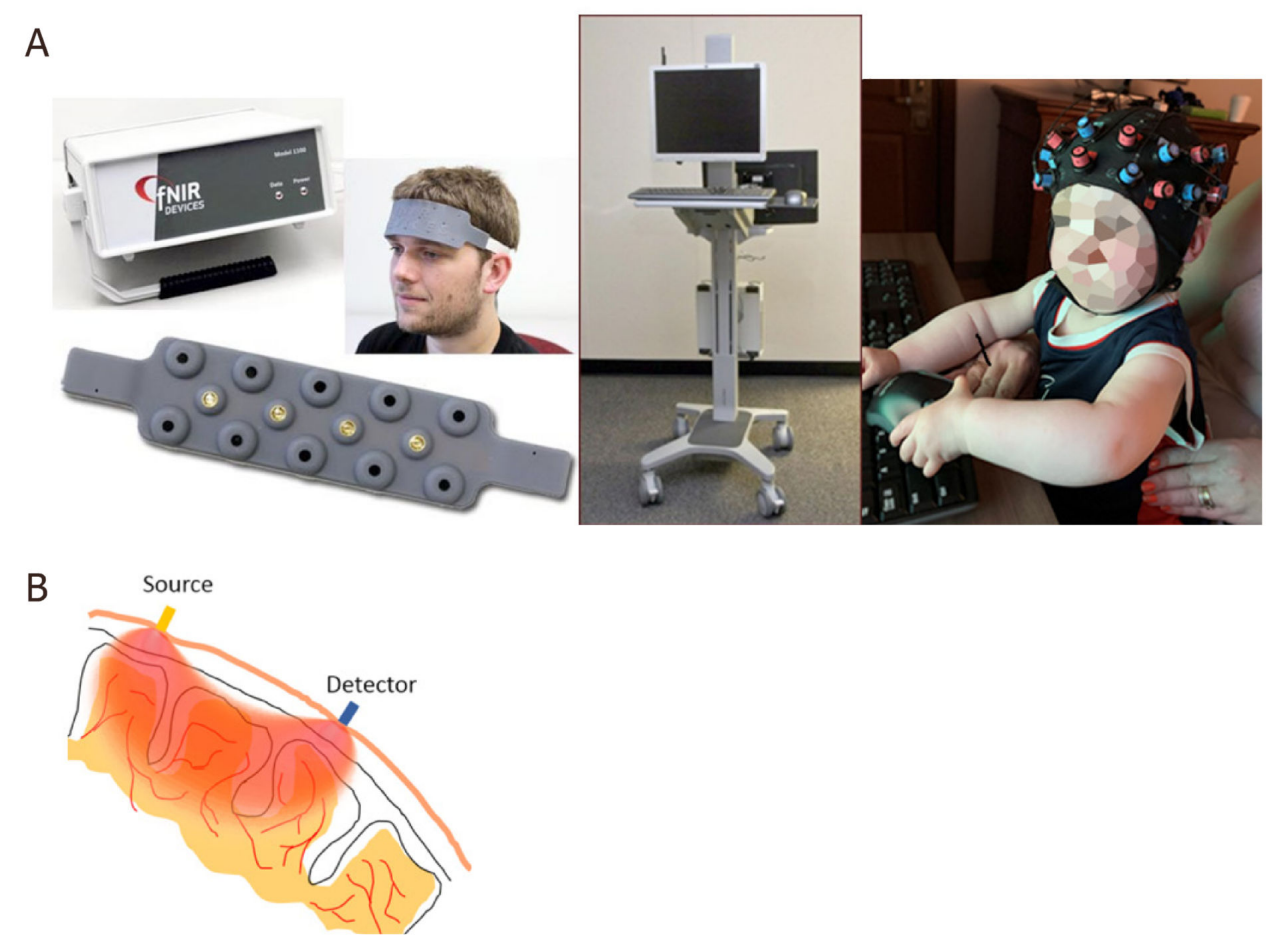
Left: the NIRS apparatus;Middle: the data-capturing device; Right: NIRS cap (NIRx NIRSPort2 Wireless system) containing sources (red marks) and detectors (blue marks) are placed on the participants’ head (A); light enters the tissue from location of source and backscattered photons are detected at the detector site (B). The detected light intensity carries information about changes in oxyhemoglobin and deoxy-hemoglobin. NIRS: near infrared spectroscopy

Although there has been no previous experience with inborn errors of metabolism, the unique features of this technique makes it ideal to evaluate brain activity in these conditions at baseline and ultimately during recovery of an acute metabolic event. Our prior fMRI experience in Urea cycle disorders has shown altered brain networks as a result of brain damage due to hyperammonemic encephalopathy^[91]^. Thus, we expected fNIRS to be a sensitive measure of brain hemodynamics in this condition and related IEMs. fNIRS may be an alternative to fMRI due to its noninvasiveness and portability. It has been used extensively in infants and children due to these advantages [Figure 3]. Currently there has not been widespread use in IEMs, but it could find potential in disorders such as PKU and galactosemia where there has been extensive work with fMRI mainly in adult populations who are able to cooperate with the scanner environment, but more recently with pediatric populations.

**Figure 4.**
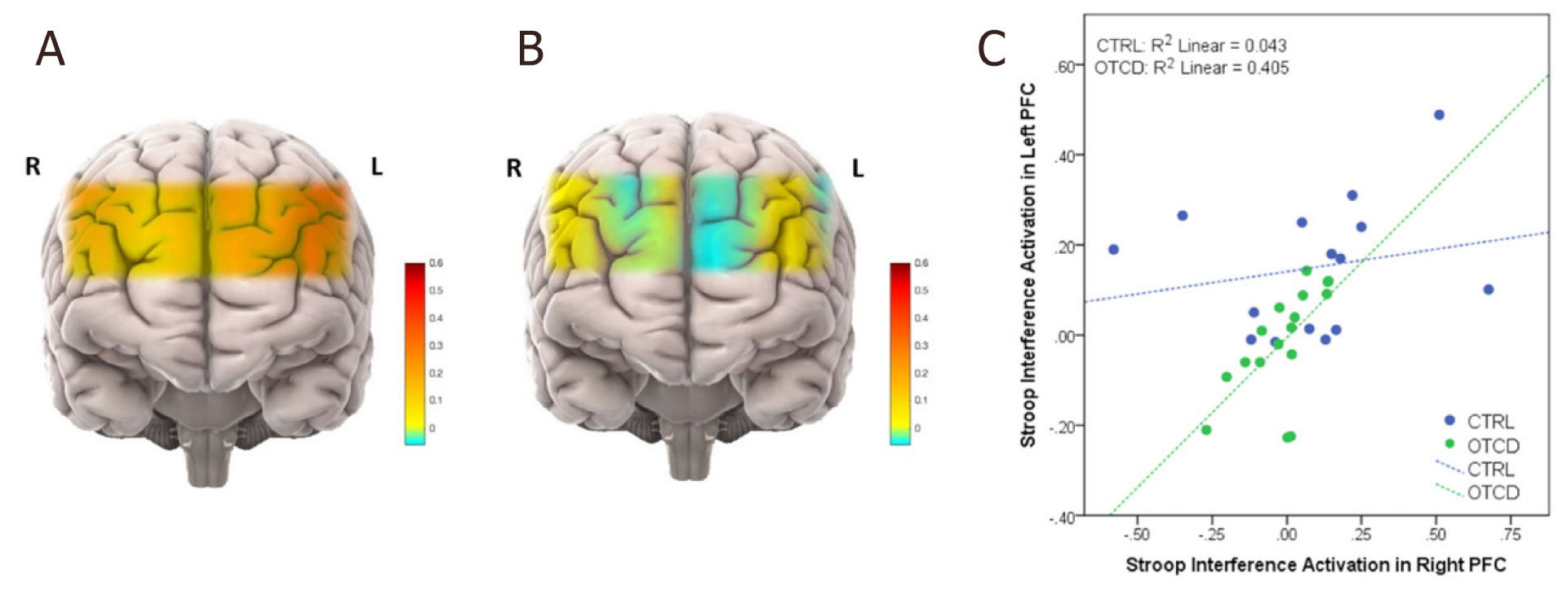
Activation maps in subjects with OTCD and controls. Prefrontal cortex activation in control (A) *vs*. OTCD cohorts indicating the lower activation during the Stroop task in OTCD group (B); stroop interference activaton in left prefrontal cortex of controls and subjects with OTCD (C). OTCD: ornithine transcarbamylase deficiency

fNIRS has also been used to investigate the biomarkers of mitochondrial and other neuro-genetic disorders^[95]^, where changes in tissue oxygen index showed greater variability among children with mitochondrial disease. Recent studies using fNIRS on prefrontal cortex (PFC) have shown that during performance of the Stroop task cohorts with OTCD showed lower left PFC activation compared to the controls. Such observation was despite the non-significant behavioral differences between the OTCD and control group. Unlike the control group, participants with OTCD also showed bilateral increase in left and right PFC, suggesting the possibility of inefficient lateralization of PFC in OTCD group [Figure 4]^[96]^.

## RESEARCH INTO CLINICAL PRACTICE

**Figure 5.**
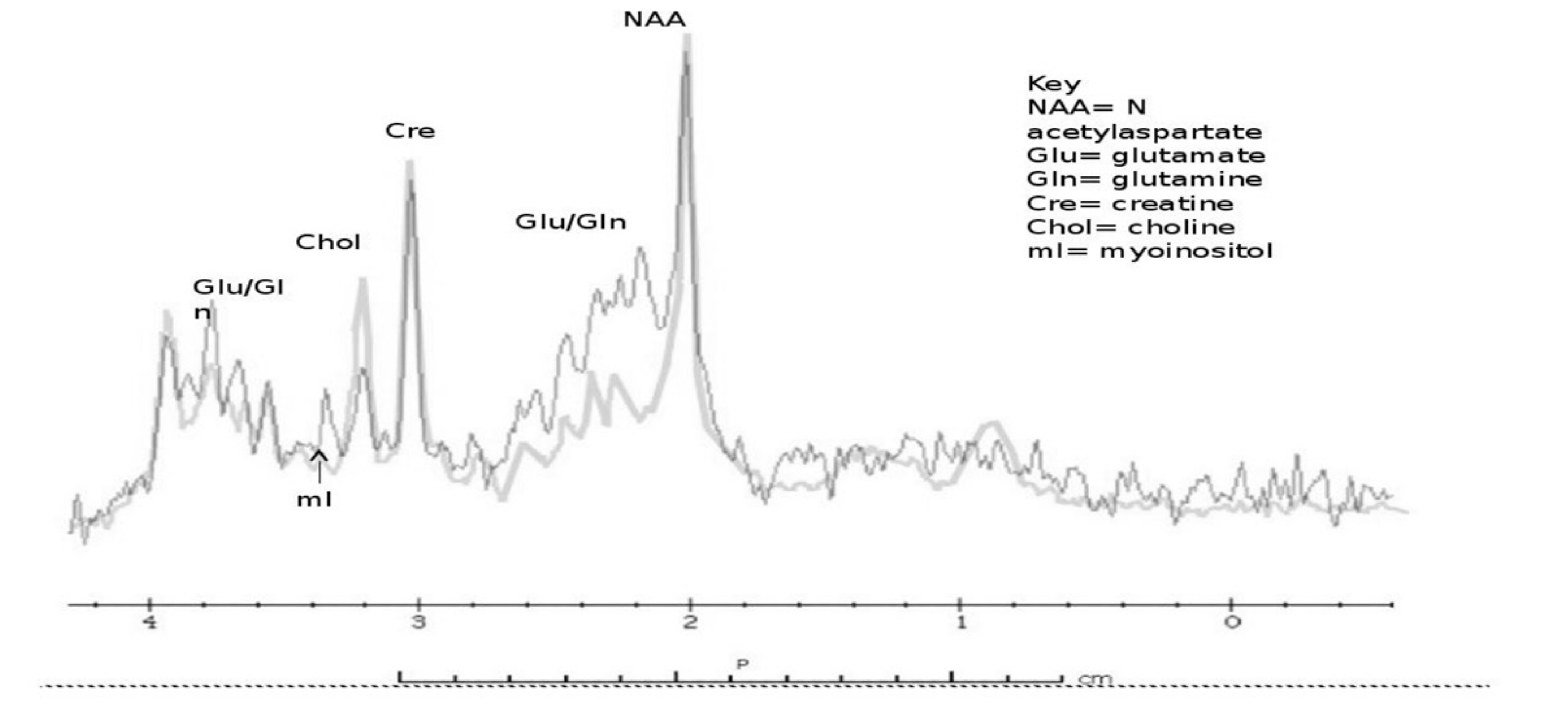
^1^H MRS showing patient with urea cycle *vs*. control subject. Single voxel MR spectroscopy (left basal ganglia) shows an elevated glutamine and glutamate peak complex and reduction of myoinositol, choline was mildly depressed. Purple: control; Black: OTCD; MRS: magnetic resonance spectroscopy; OTCD: ornithine transcarbamylase deficiency

How does research neuroimaging impact on clinical practice? Our research in the UCDC has already allowed research into practice. As we have shown, Urea cycle-related disorders may show variable neuroimaging manifestations, ranging from normal to abnormal with or without a signature appearance. In the past, we have described the role of multimodal imaging in identifying biomarkers of neuronal injury in UCD patients. The diagnosis by clinical features and MRS was much faster than pending (in this case would have been next day) plasma amino acids and urine organic acids results. This research was put into clinical contact recently when we were able to achieve a rapid diagnosis and treatment of a 3 year old boy with mental status changes suspected due to new onset OTCD due to a high index of suspicion and classic findings on MR spectroscopy [Figure 5]. This case demonstrated the practicality of MR spectroscopy in discerning OTCD from other inborn errors of metabolism causing hyperammonemia; which can have a crucial impact on the acute course of events during the initial presentation when confirmatory testing has not yielded results.

**Figure 6.**
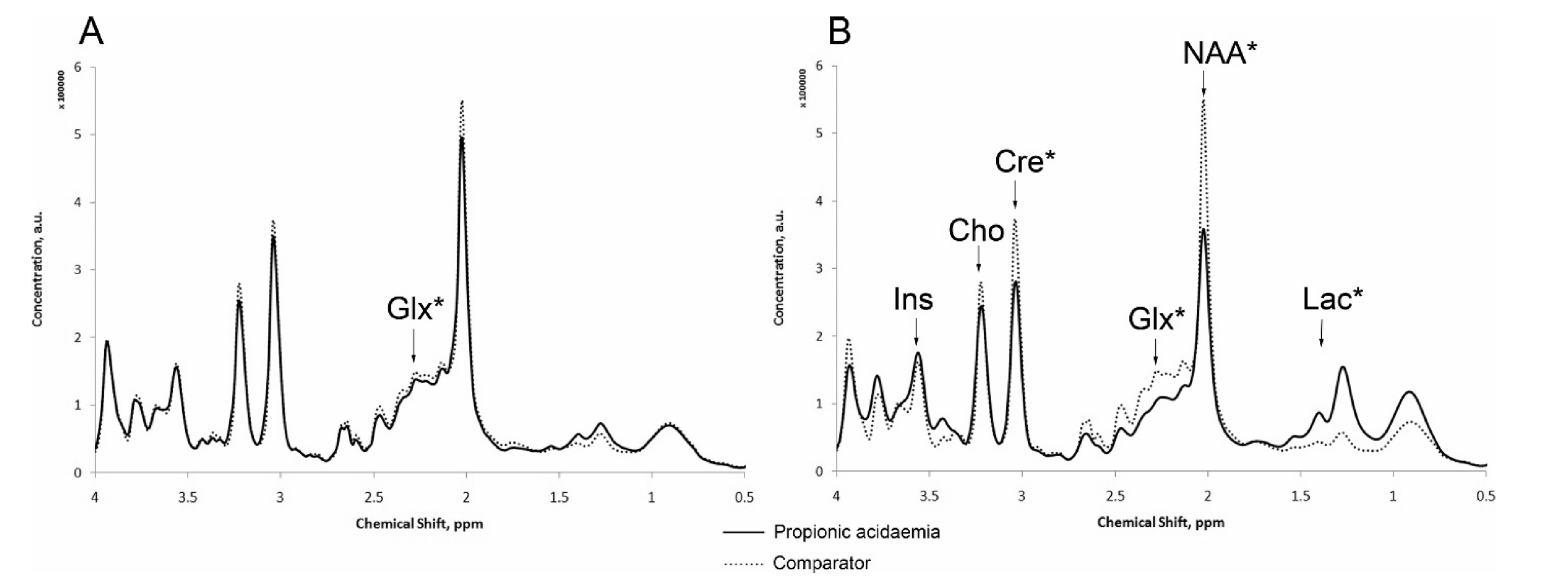
MR spectra from basal ganglia during metabolic stability and acute encephalopathy in propionic acidemia. Glx: glutamine + glutamate; Ins: myo-inositol; Cre: Creatine; NAA: N-acetylaspartate; Lac: lactic acid

Davison *et al*.^[97]^ used Quantitative MRS to evaluate concentrations of brain metabolites in children with neonatal onset propionic acidemia. They tracked metabolic stability, during an acute encephalopathic episode prior to and after liver transplantation. They concluded that MRS was useful in this setting and believed it could also be used to evaluate potential therapies [Figure 6].

In the last decade, advances in neuroimaging technologies have led to multiple studies investigating the neural networks of executive function (EF). EF has long been associated with the PFC involving working memory, inhibitory control and set shifting. Diamond speculated that neuroimaging can allow the investigation of many unanswered questions regarding cognitive functions and stated that fMRI and fNIRS could be of interest in studying the neural correlates of cognition. There are ample opportunities to employ this strategy in IEMs^[98]^.

## CONCLUSION

MRI imaging beyond routine structural imaging can shed light on processes that impact the brain during metabolic crisis. Collaboration with physicists and neuroimaging specialists can allow further detailed study and identify biomarkers that can be quickly put into clinical practice.

## Figures and Tables

**Table 1.** ^1^H MRS findings in select inborn errors of metabolism

	NAA	Cho	MI	Glx	lac	Other peaks

Zellweger	↓	↑	↓	↑	↑	Lipid
Neonatal ALD	↓	↑				Lipid
Infantile Refsum	↓	↑	↑			Lipid
RCDP		↓	↑			Lipid, acetate
PDH	↓				↑	Acetate
NKH						Glycine
S-L-O		↑				Lipid
Salla	↑	↓				
CDG	↓	↓	↑	↑		
CPS1, OTCD		↓	↓	↓	↔	
GA type 1	↓	↑	↑	↑		
GA type 2		↑				
Mucolipidoses	↓					
Krabbe	↓	↑	↑	↑	↑	
MPS	↓	↑	↑			
MMA	↓				↑	
ALD		↓				
Arginase		↔	nl	↑		

The arrows indicate the direction of the change. ↓: decreased; ↑: increased; ↔: no change. ALD: adrenoleukodystrophy; RCDP: rhizomelic chondrodysplasia punctate; PDH: pyruvate dehydrogenase deficiency; KNH: nonketotic hyperglycinemia; SLO: Smith Lemli Opitz; CDG: congenital disorders of glycosylation; CPS1: carbomyl phosphate synthetase deficiency; OTCD: ornithine transcarbamylase deficiency; GA: glutaric acidemia; MPS: mucopolysacharoidosis; MMA: methylmalonic acidemia; nl: normal
